# Klotho-derived peptide 1 ameliorates hepatic fibrosis induced by αKlotho deficiency and liver injury

**DOI:** 10.7150/ijbs.122107

**Published:** 2026-01-08

**Authors:** Huishi Tan, Wenshu Huang, Hanying Luo, Wenjian Min, Xiaoyao Zhang, Xiaoli Sun, Enqing Lin, Xue Hong, Peng Yang, Lili Zhou, Youhua Liu

**Affiliations:** 1 State Key Laboratory of Multi-organ Injury Prevention and Treatment, National Clinical Research Center for Kidney Disease, Division of Nephrology, Nanfang Hospital, Southern Medical University, Guangzhou, China.; 2 Guangdong Provincial Key Laboratory of Renal Failure Research, Guangdong Provincial Institute of Nephrology, Guangzhou, China.; 3 State Key Laboratory of Natural Medicines and Jiangsu Key Laboratory of Drug Design and Optimization, China Pharmaceutical University, Nanjing, China.

**Keywords:** Klotho, TGF-β receptor 2, liver fibrosis, KP1, TGF-β, hepatic stellate cells

## Abstract

Hepatic fibrosis, driven primarily by hepatic stellate cells (HSCs) activation induced by TGF-β, currently lacks effective therapies. In this study, we demonstrated that deficiency of αKlotho, an extrahepatic antiaging protein, due to genetic ablation in *kl/kl* model or aging caused spontaneous hepatic fibrosis, as evidenced by an increased collagens deposition and TGF-β signaling hyperactivation. KP1, a small peptide derived from human αKlotho protein, recapitulated its anti-fibrotic potential and blocked HSCs activation induced by TGF-β1. Mechanistically, KP1 acted as a competitive TGF-β receptor 2 (TβR2) antagonist, disrupted TGF-β1/TβR2 engagement, and suppressed both canonical and noncanonical TGF-β signaling in HSCs. Infusion of KP1 *in vivo* rescued hepatic integrity, restored liver function, inhibited TGF-β signaling and mitigated hepatic fibrosis in *kl/kl* mice. In mouse model of carbon tetrachloride-induced hepatic fibrosis, KP1 exhibited preferential accumulation in injured liver after intravenous injection, disrupted TGF-β1/TβR2 interaction, inhibited HSCs activation, and ameliorated hepatic fibrosis. Similarly, KP1 also mitigated cholestatic fibrosis induced by bile duct ligation. Collectively, these studies establish KP1 as a novel, mechanism-driven therapeutic peptide that potently inhibits HSCs activation and liver fibrogenesis. Its liver-targeted delivery and efficacy across diverse fibrosis models underscore KP1 as a promising next-generation therapeutic remedy for fibrotic liver disease.

## Introduction

Liver fibrosis, characterized by excessive deposition of extracellular matrix (ECM) leading to tissue scarring, is the common outcome of a wide variety of chronic liver disease, regardless of the initial etiologies 1-4. The fibrotic lesions in the liver, if untreated, often progress and culminate in irreversible cirrhosis and liver failure with a high rate of morbidity and mortality, underscoring an urgent need for effective therapies 5-7. The central event during liver fibrosis is the activation of hepatic stellate cells (HSCs) and portal fibroblasts, which transform from quiescent state into proliferative, contractile, ECM-producing myofibroblasts 8, 9. Among many factors controlling HSCs activation, transforming growth factor-β (TGF-β) is the chief one that is responsible for driving HSCs activation and ECM overproduction after liver injury 10-13.

TGF-β transduces its signal by initially binding to cell membrane TGF-β type II receptor (TβR2), which then recruits and phosphorylates the type I receptor (TβR1). This leads to subsequent phosphorylation and activation of Smad-2 and Smad-3 transcription factors and promotes the expression of various fibrosis-related genes 14-16. Meanwhile, TGF-β also stimulates Smad-independent, non-canonical pathways, particularly the mitogen-activated protein kinases (MAPKs) including extracellular signal-regulated protein kinese-1 and -2 (ERK1/2), c-Jun N-terminal kinase (JNK), and p38 MAPK, further amplifying profibrotic responses 13, 17. TGF-β signaling is tightly controlled *in vivo* by endogenous antagonists such as αKlotho, an anti-aging protein predominantly produced by kidney tubular epithelial cells 18, 19. These findings point to a potential connection between hepatic fibrosis and aging or αKlotho deficiency. However, the exact relationship and interplay among aging, αKlotho depletion, and liver fibrosis remain to be explored.

αKlotho exists as both a transmembrane protein and a circulating soluble form (sKlotho) 20-23. Apart from its established function as an essential co-receptor for endocrine fibroblast growth factor 23 (FGF23) in regulating diverse physiological processes ranging from mineral metabolism to energy homeostasis, αKlotho exhibits potent anti-fibrotic activity across different organs, primarily by antagonizing TGF-β, Wnt, and FGF2 signaling 18, 24-27. However, the therapeutic utility of αKlotho is hampered by its large size, expensive to produce, and potential undesirable effect associated with hypophosphatemia 28. We recently discovered a Klotho-derived peptide (KP1), which recapitulates the anti-fibrotic action of αKlotho by inhibiting TGF-β signaling 19. KP1 may offer distinguished advantages over αKlotho, such as high efficacy, reliable safety, well tolerability, and easy to chemically produce. However, it remains unknown whether KP1 can rescue hepatic integrity in αKlotho deficiency and ameliorate fibrotic lesions after liver injury.

In this study, we showed that aging or αKlotho deficiency spontaneously induces hepatic fibrosis in mice. By molecular docking simulation and site-directed mutations, we have defined the key amino acids in KP1 that is responsible for mediating KP1/TβR2 interaction. More importantly, we demonstrated that KP1 can rescue hepatic integrity and restore liver function in genetic αKlotho-deficient mice, and in mouse models of liver injury and fibrosis induced by carbon tetrachloride (CCl₄) or bile duct ligation (BDL), respectively. These findings identify KP1 as a novel and effective small-molecule remedy that exhibits potent anti-fibrotic efficacy in preclinical setting.

## Results

### Aging or αKlotho deficiency spontaneously induces hepatic fibrosis

We found that aged mice exhibited an impaired hepatic function, an increased expression and deposition of extracellular matrix (ECM) and an enhanced cellular senescence in the liver (Figure S1). Comparing to young mice at 2 months, circulating levels of alanine aminotransferase (ALT) and aspartate aminotransferase (AST) elevated in old mice at age of 21 months (Figure S1A and B). Hepatic expression of fibronectin and α-smooth muscle actin (α-SMA) was upregulated (Figure S1C-F). Meanwhile, cellular senescent markers such as p16, p53, p19, and phosphorylated H2AX (γH2AX) were induced in the liver of old mice (Figure S1G-I). Notably, these hepatic changes in old mice were accompanied by the loss of αKlotho in the kidneys (Figure S1J and K), suggesting a possible connection between liver aging and αKlotho deficiency in the kidney.

**Figure 1:**
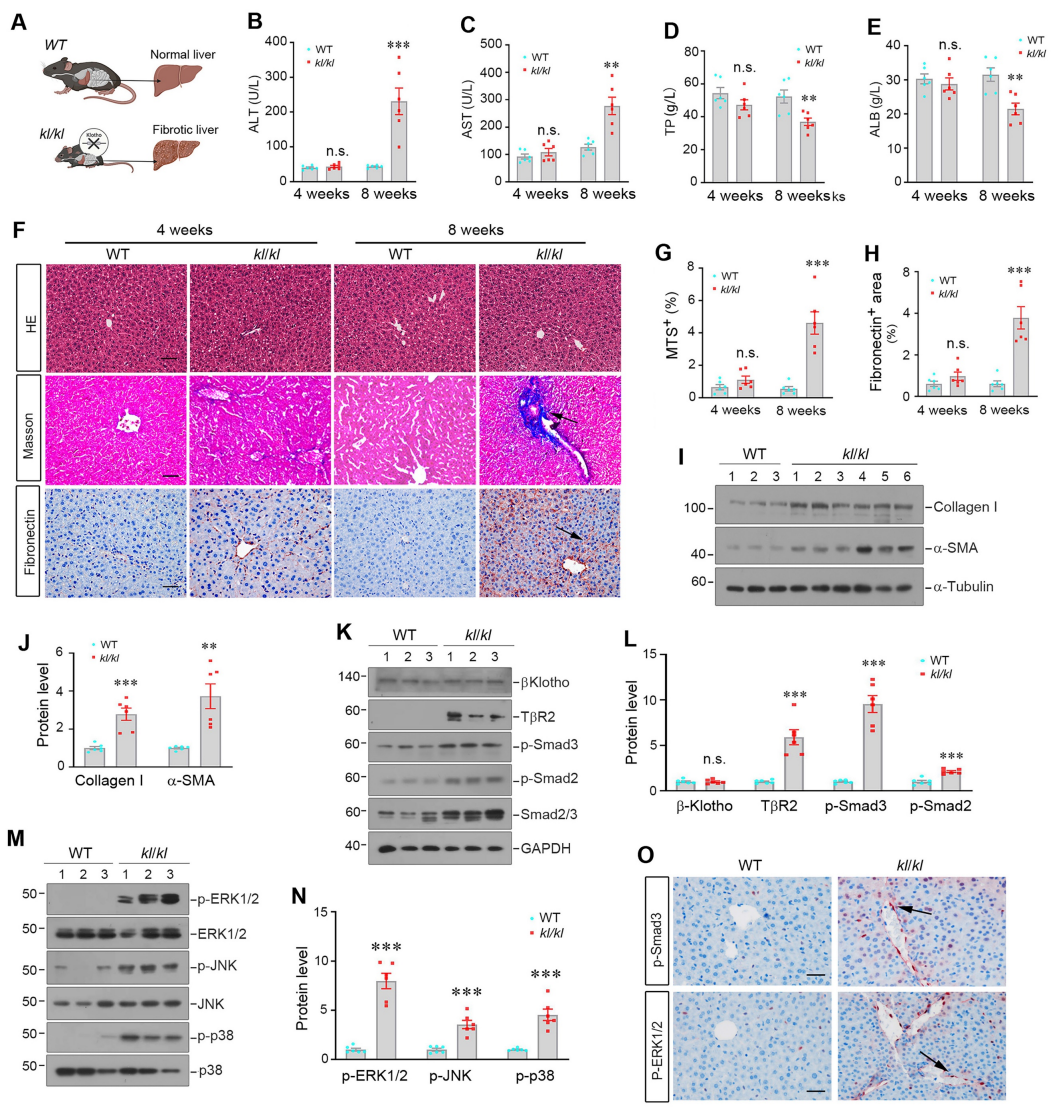
Deficiency of αKlotho causes hepatic fibrosis by activating TGF-β signaling. (A) Schematic presentation of WT and *kl/kl* mice. (B-E) Plasma levels of alanine aminotransferase (ALT), aspartate aminotransferase (AST), total protein (TP), and albumin (ALB) in WT and *kl/kl* mice at 4 and 8 weeks, respectively (n=6). (F) Representative micrographs of H&E, Masson's trichrome staining (collagen, blue), and fibronectin immunohistochemical staining in liver sections. Scale bar: 50 µm. (G, H) Quantification of Masson's trichrome staining (MTS) and fibronectin-positive area in different groups as indicated. (I, J) Representative immunoblots (I) and quantitative data (J) of α-SMA and collagen Ⅰ in liver lysates in different groups as indicated. (K, L) Representative immunoblots (K) and quantitative data (L) of TβR2, β-klotho, p-Smad2, p-Smad3, and Smad2/3 in different groups as indicated. (M, N) Representative immunoblots (M) and quantitative data (N) of p-ERK1/2, p-JNK, p-p38, and corresponding total proteins. (O) Representative micrographs of p-Smad3 and p-ERK1/2 in the livers of WT and *kl/kl* mice. Scale bar, 50 µm. ^**^P < 0.01 versus WT, ^***^P < 0.001 versus WT (n=6).

To establish the potential role of αKlotho in liver homeostasis, aging and fibrosis, we utilized the genetic αKlotho-deficient (*kl/kl*) mice (Figure 1A and Figure S2A). As shown in Figure 1B-E, compared to wild-type (WT) littermates, 8-week-old *kl/kl* mice exhibited liver dysfunction, evidenced by elevated ALT and AST in the circulation, and reduced serum albumin (ALB) and total protein (TP) (Figure 1B-E). Histological assessment revealed pronounced collagen deposition and increased fibronectin immunostaining in *kl/kl* livers (Figure 1F-H). Immunoblotting confirmed the upregulation of α-smooth muscle actin (α-SMA) and collagen I in liver homogenates of *kl/kl* mice, compared with WT controls (Figure 1I-J), suggesting that loss of αKlotho spontaneously induces fibrotic lesions in the liver.

We next investigated the potential mechanism underlying liver fibrosis in *kl/kl* mice. Notably, αKlotho was undetectable in the liver of both *kl/kl* and WT mice, as shown by Western blot analysis (Figure S2B and C). We found that despite the loss of αKlotho, βKlotho expression remained unchanged in the livers of* kl/kl* mice (Figure 1K and L). However, loss of αKlotho resulted in hyperactivation of TGF-β signaling, as TβR2 expression was upregulated (Figure 1K and L). Consistently, TGF-β downstream signaling effectors such as Smad2/Smad3, ERK1/2, JNK, and p38 MAPK were phosphorylated and activated in *kl/kl* livers (Figure 1K-N). Immunohistochemical staining for p-Smad3 and p-ERK revealed an increased expression of these signaling effectors primarily in non-parenchymal cells such as HSCs or fibroblasts (Figure 1O). In contrast, Wnt/β-catenin and FGF2 signaling was unaffected in the livers of *kl/kl* mice (Figure S3). These results suggest that loss of αKlotho due to aging or genetic ablation causes hepatic fibrosis primarily via activating TGF-β signaling.

### Molecular docking and mutations define the binding site of KP1 to TβR2

**Figure 2:**
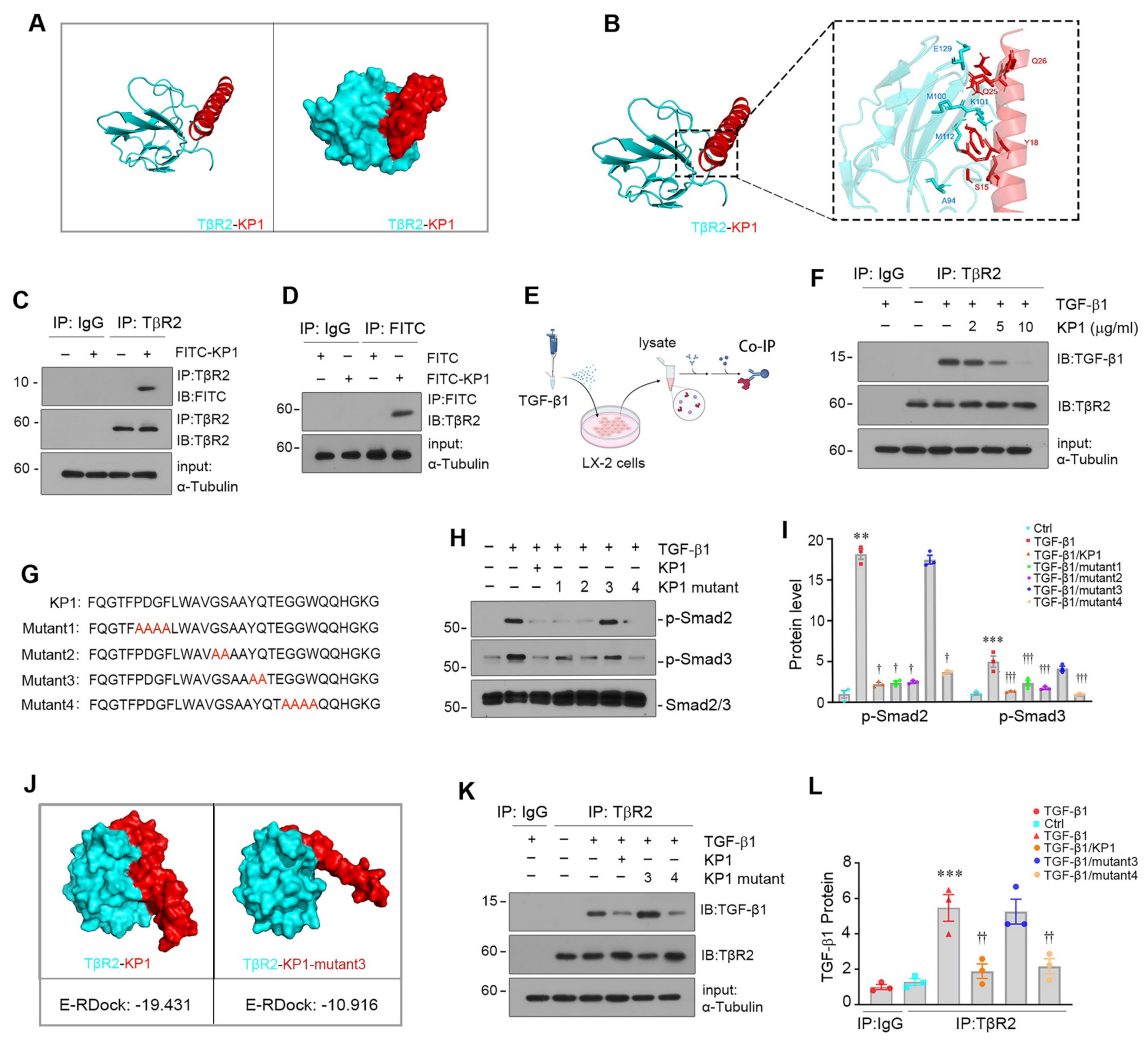
Molecular docking simulation and site-directed mutation identify key amino acids of KP1 mediating interaction with TGF-β receptor 2. (A, B) Molecular docking simulation predicted the binding sites between KP1 and TβR2 (PDB ID:1M9Z). The 3D model (A) and detailed contact sites (B) are shown. (C, D) Co-immunoprecipitation of TβR2 and FITC-KP1. The LX-2 cell lysates were incubated with FITC-KP1, and immunoprecipitated with antibodies against TβR2 (C) or FITC (D), respectively, followed by immunoblotted with TβR2 or FITC antibodies. (E) Schematic diagram of the experimental design. LX-2 cells were pre-incubated with KP1 for 1 h, then treated with TGF-β1 (2 ng/ml) for 5 min. Cell lysates were immunoprecipitated with anti-TβR2 or anti-IgG. (F) Co-immunoprecipitation of TβR2 with TGF-β1 in LX-2 cells. KP1 dose-dependently inhibited TGF-β1 binding to TβR2. (G) The sequence of KP1 and its variants. Amino acids marked in red represent the sites of mutation in KP1. (H, I) Representative immunoblot (H) and quantitative data (I) show the levels of p-Smad2 and p-Smad3 in different groups as indicated. (J) Molecular docking simulations show the difference between KP1/TβR2 and KP1-mutant3/TβR2 binding. The E-RDOCK scores were presented at the bottom of the images, respectively. (K, L) Co-immunoprecipitation showed the effect of KP1 and its variants on TGF-β1 and TβR2 interaction. Representative blot (K) and quantitative data (L) are presented. ^**^P < 0.01,^***^P < 0.001 versus controls; ^†^*P* < 0.05, ^††^*P* < 0.01, ^†††^*P* < 0.001 versus TGF-β1 (n=3).

We previously reported the discovery of KP1, a small peptide derived from human αKlotho (Phe57-Gly86) that binds to TβR2 and inhibits TGF-β signaling 19. To further characterize the key amino acid of KP1 responsible for binding to TβR2, we employed molecular docking simulation technique to define KP1-TβR2 interaction sites. As shown in Figure 2A and B, KP1 exhibited high-affinity binding to TβR2 (PDB ID:1M9Z), with S15 and Y18 as contact sites, which correspond to Ser71 and Tyr74 in human αKlotho, respectively. Notably, co-immunoprecipitation (Co-IP) analysis revealed that FITC-tagged KP1 physically interacted with TβR2 in human stellate cells (LX-2) (Figure 2C and D), validating the binding of KP1 to TβR2. We found that KP1 competed with TGF-β1 for binding to TβR2, as it does-dependently impeded TGF-β1 and TβR2 interaction (Figure 2E and F).

KP1 sequence showed high evolutionary conservation across different species 19. To functionally validate the role of key amino acids of KP1 for binding to TβR2, we synthesized four KP1 variants with mutations at different locations by substituting the corresponding amino acid with alanine (Figure 2G), respectively. As shown in Figure 2H and I, alanine mutation scanning revealed that KP1-mutant3, with Y18A/Q19A mutations, failed to suppressed TGF-β1-mediated Smad2/3 activation. We further utilized molecular docking simulation to predict its interaction with TβR2. As shown in Figure 2J, Y18A/Q19A mutations in KP1-mutant3 rendered it unable to bind to TβR2 with a high affinity. Consistently, Co-IP studies confirmed KP1-mutant3 lost the ability to disrupt TGF-β1/TβR2 interaction (Figure 2K and L). Therefore, the Y18/Q19 in the KP1, which are completely conserved evolutionarily from human to *Drosophila* 19, are the key amino acids responsible for its binding to TβR2.

### KP1 inhibits hepatic stellate cell activation *in vitro*

**Figure 3:**
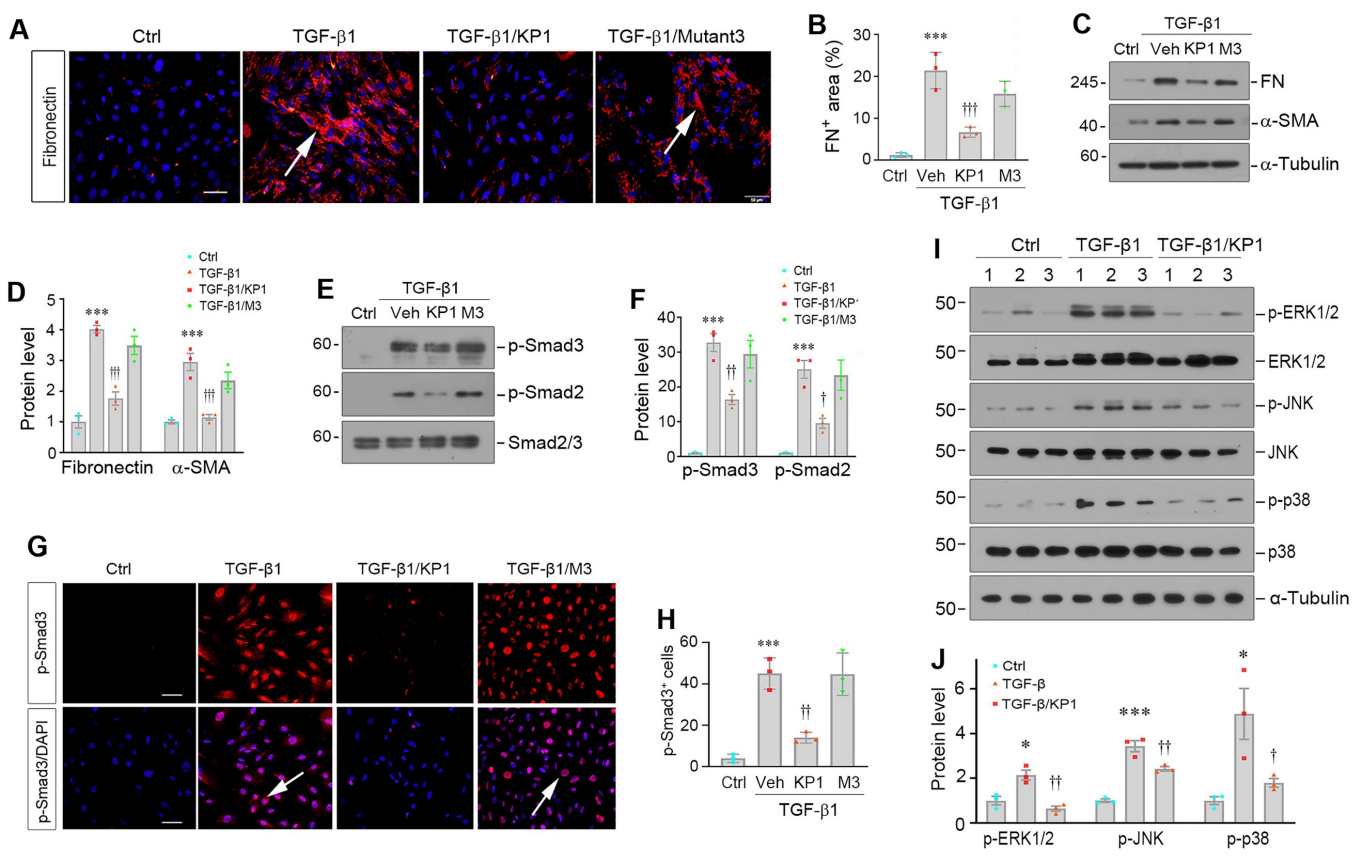
KP1 suppresses TGF-β1-induced myofibroblastic activation of hepatic stellate cells. (A, B) Representative micrographs (A) and quantitative data (B) show fibronectin expression in hepatic stellate cells (LX-2). The LX-2 cells were pre-incubated with KP1 or KP1-Mutant3 (10 µg/ml; 3 µM) for 1 h and then treated with TGF-β1 (2 ng/ml) for 24 h. Scale bar, 50 µm. Arrows indicate positive staining. (C, D) Representative Western blot (C) and quantitative data (D) show that KP1 inhibited the expression of fibronectin (FN) and α-smooth muscle actin (α-SMA) induced by TGF-β1 in LX-2 cells. (E, F) Representative Western blot (E) and quantitative data (F) show the expression of levels of p-Smad2 and p-Smad3 in different groups as indicted. (G, H) Representative micrographs (G) and quantitative data (H) show Smad3 phosphorylation and nuclear translocation in LX-2 cells after different treatments as indicated. Serum-starved LX-2 cells were pre-incubated with KP1 or KP1-Mutant3 for 1 h and then treated with TGF-β1 (2 ng/ml) for 45 min. DAPI denotes the nuclei. Scale bar, 50 µm. Arrows indicate p-Smad3 positive cells. (I, J) Representative immunoblots (I) and quantitative data (J) of p-ERK1/2, p-JNK, p-p38, and corresponding total proteins. ^*^P < 0.05, ^***^P < 0.001 versus controls; ^†^*P* < 0.05, ^††^*P* < 0.01, ^†††^*P* < 0.001 versus TGF-β1 (n=3). Veh, vehicle, M3, KP1-Mutant3.

We found that KP1 was able to inhibit HSCs activation induced by TGF-β1 *in vitro*. Incubation of LX-2 cells with TGF-β1 induced fibronectin expression and deposition, which was abolished by KP1, but barely by KP1-mutant3 (Figure 3A and B). Similarly, Western blot analyses showed that KP1, but not KP1-mutant3, blocked TGF-β1-induced α-SMA and fibronectin expression at protein levels (Figure 3C and D). KP1, but not KP1-mutant3, abolished TGF-β1-induced Smad2 and Smad3 phosphorylation and nuclear translocation in LX-2 cells (Figure 3E-H). KP1 concurrently suppressed the ERK1/2, JNK, and p38 MAPK activation induced by TGF-β1 in LX-2 cells (Figure 3I and J), suggesting that KP1 is able to block both Smad-dependent, canonical- and Smad-independent, noncanonical TGF-β signaling *in vitro*. However, neither αKlotho nor KP1 exerted any significant influence on the protein expression levels of α-SMA, collagen I, or fibronectin in LX-2 cells in the absence of TGF-β1 stimulation, suggesting they do not affect HSC activation under basal condition (Figure S4A and B).

### KP1 rescues hepatic integrity and restores liver function in αKlotho null mice

**Figure 4:**
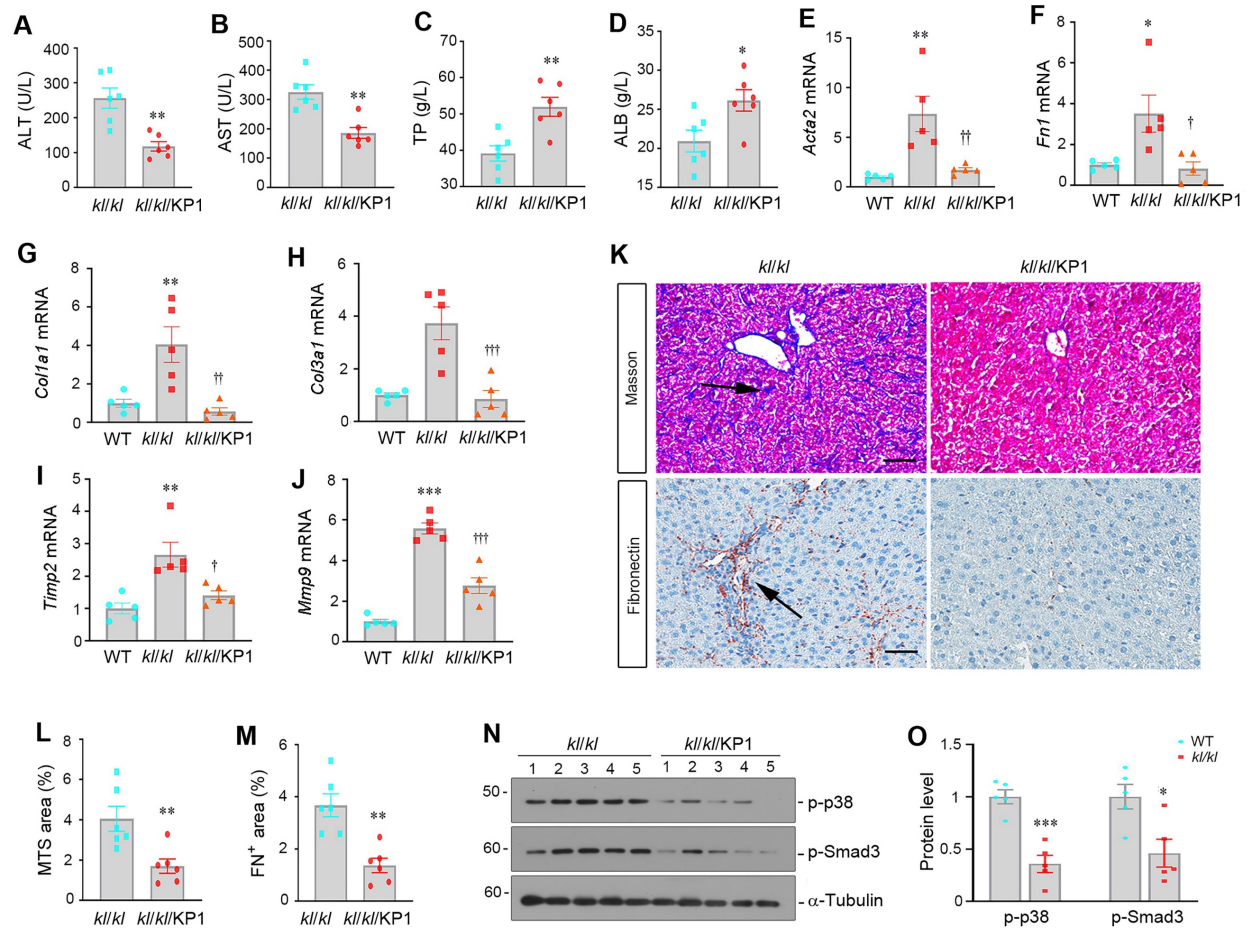
KP1 reverses α-Klotho deficiency-induced hepatic fibrosis. (A-D) KP1 reduced serum levels of alanine aminotransferase (ALT) and aspartate aminotransferase (AST) but increased total protein (TP) and albumin (ALB) levels in *kl/kl* mice. (E-J) KP1 inhibited hepatic mRNA expression of multiple profibrotic genes in *kl/kl* mice. Liver mRNA levels of *Acta2, Fn1, Col1a1, Col3a1, Timp2,* and* Mmp9* genes were assessed by quantitative, real-time RT-PCR (RT-qPCR). (K-M) Representative micrographs and quantitative data of Masson's trichrome staining (K, L) and immunohistochemical staining for fibronectin (K, M). Scale bar, 50 µm. (N, O) Representative Western blot (N) and quantitative data (O) show that KP1 inhibited Smad3 and p38 MAPK phosphorylation and activation in *kl/kl* mice. Liver extracts were immunoblotted with antibodies to p-p38, p-Smad3, and α-tubulin, respectively. ^*^P < 0.05, ^**^P < 0.01, ^***^P < 0.001 versus WT; ^†^*P* < 0.05, ^††^*P* < 0.01, ^†††^*P* < 0.001 versus *kl/kl* (n=5-6).

To test whether KP1 can mimic endogenous αKlotho in preventing hepatic fibrosis, we administered KP1 using ALZET osmotic pumps for 4 weeks to *kl/kl* mice, starting at 4 weeks of age. As shown in Figure 4A-D, KP1 normalized serum ALT and AST levels and partially restored ALB and TP levels in *kl/kl* mice. KP1 also inhibited hepatic mRNA expression of numerous fibrosis-related genes such as α-smooth muscle actin (*Acta2*), *Fn1*, *Col1a1*, *Col3a1*, tissue Inhibitor of metalloproteinase 2 (*Timp2*), and matrix metalloproteinase-9 (*Mmp9*) (Figure 4E-J). Masson's trichrome staining for collagens and immunohistochemical staining for fibronectin revealed that KP1 reduced collagens and fibronectin deposition (Figure 4K-M). Western blotting confirmed that KP1 repressed Smad3 and p38 MAPK phosphorylation and activation in the liver of *kl/kl* mice (Figure 4N-O). These results suggest that KP1 recapitulates the anti-fibrotic action of αKlotho by inhibiting TGF-β signaling and rescues normal liver phenotype in αKlotho null mice.

### KP1 targets injured liver and ameliorates hepatic fibrosis induced by carbon tetrachloride

**Figure 5:**
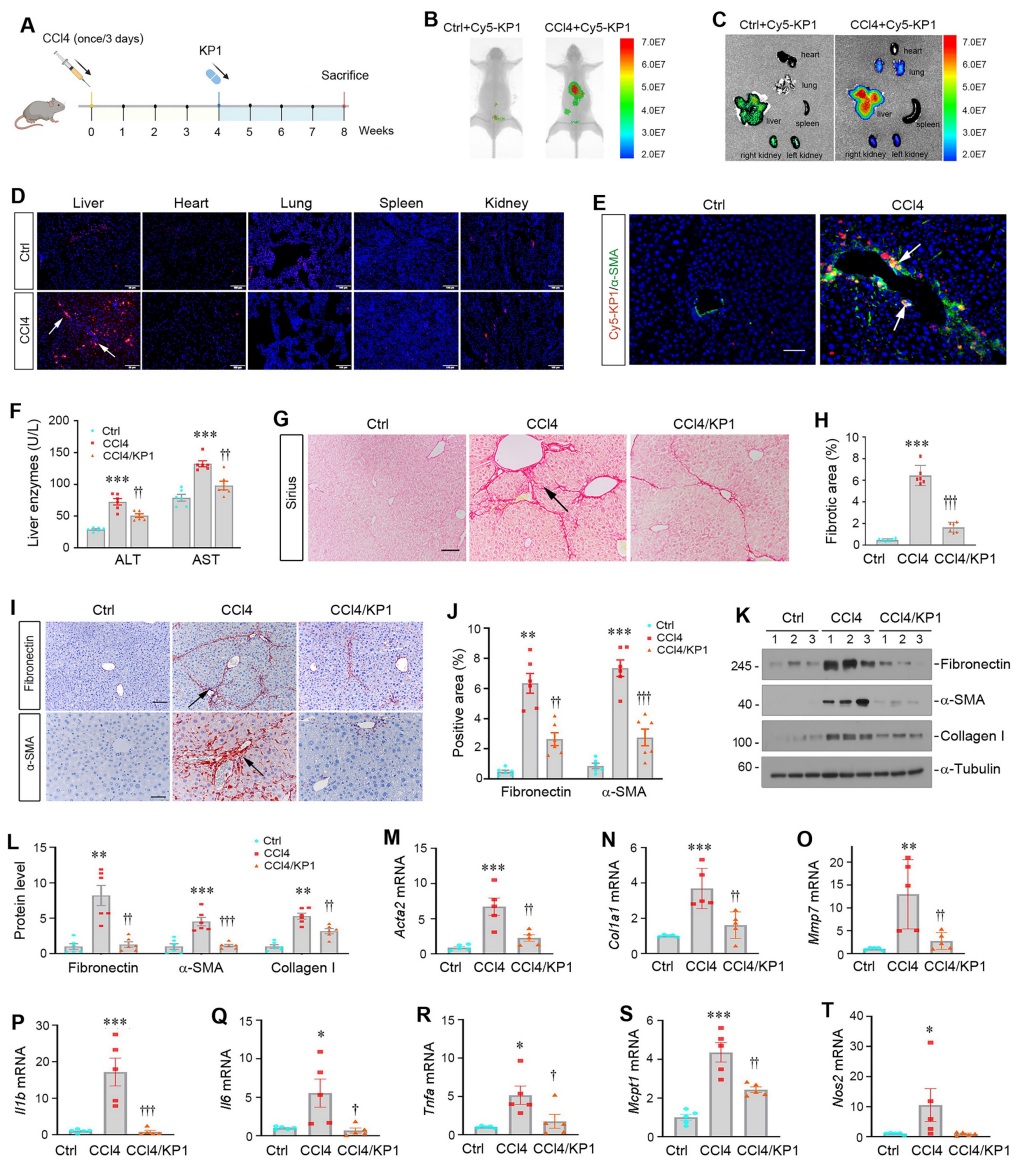
KP1 ameliorates CCl_4_-induced liver injury and fibrosis in mice. (A) Schematic diagram of the experimental design. Mice were injected with CCL_4_ for 8 weeks to induce liver injury and fibrosis. At 4 weeks, mice were randomly divided into KP1 (1 mg/day/kg body weight) or vehicle (0.01M acetic acid) groups. The blue color indicates the duration of KP1 infusion. (B-C) Organ imaging showed that KP1 was preferentially accumulated in the injured liver after CCl_4_ injections. Relative levels of Cy5-KP1 are shown in major organs. (D) Representative micrographs of Cy5-KP1 distribution in different organs. Control (Ctrl) or CCl_4_ mice were sacrificed 0.5 h after intravenous injection of Cy5-KP1. Tissue cryosections were observed for fluorescent expression by a laser confocal microscope. Scale bar, 50 µm. (E) Colocalization showed that Cy5-KP1 could target the α-SMA^+^ myofibroblasts in the fibrotic liver after CCl_4_ injections. (F) Plasma ALT and AST levels in CCl_4_ mice with or without KP1 treatment. (G) Representative micrographs of Sirius red staining in different groups as indicated. Scale bar, 50 µm. The arrow indicates collagen deposition. (H) Quantitative determination of hepatic fibrotic lesions. (I) Representative micrographs of immunohistochemical staining for fibronectin and α-SMA. Scale bar, 50 µm. (J) Quantitative data of fibronectin- and α-SMA-positive area in each high-power field. (K, L) Representative Western blot (K) and quantitative data (L) of hepatic fibronectin, α-SMA, and collagen Ⅰ in different groups as indicated. Numbers (1, 2, and 3) indicate each animal in the given group. (M-O) KP1 inhibited the mRNA expression of multiple fibrosis-related genes. RT-qPCR demonstrated the mRNA expression of *Acta2, Col1a1,* and* Mmp7* in the liver of different groups as indicated. (P-T) KP1 inhibited proinflammatory cytokine expression. Hepatic *Il1b, Il6, Tnfa, Mcpt1,* and* Nos2* mRNA expression was assessed by RT-qPCR. ^*^P < 0.05, ^**^P < 0.01, ^***^P < 0.001 versus controls; ^†^*P* < 0.05, ^††^*P* < 0.01, ^†††^*P* < 0.001 versus CCl_4_ (n=5-6).

We first investigated the tissue distribution of exogenous KP1 after intravenous injection. To this end, mice were subjected to CCl_4_ injections (Figure 5A). Four weeks later, control or CCl_4_-treated mice were intravenously injected with Cy5-labeled KP1. The distribution of Cy5-labeled KP1 was assessed at 30 min after injection with Bruker FX PRO imaging system. As shown in Figure 5B, Cy5-labeled KP1 was primarily and preferentially accumulated in the injured liver of the CCl_4_-treated mice, compared to control mice. Major organs including liver, kidneys, heart, lungs and spleen were removed and assessed for Cy5-labeled KP1 accumulation, which gave rise to similar results (Figure 5C). Frozen sections of major organs also showed that Cy5-labeled KP1 was predominantly localized in the injured liver of CCl_4_-treated mice (Figure 5D), in which it was largely co-localized with α-SMA (Figure 5E). Notably, the expression of TβR2 was substantially and specifically increased in the livers of CCl_4_-treated mice, compared to the controls (Figure S5A and B).

We then investigated the therapeutic role of KP1 in hepatic fibrosis by using a model with established liver injury at 4 weeks after CCl_4_ injections (Figure 5A). As shown in Figure 5F, infusion of KP1 starting at week 4 decreased serum ALT and AST levels at 8 weeks after CCl_4_ injections. Hematoxylin-eosin (HE) staining demonstrated that KP1 reduced pseudolobule formation, hepatocyte ballooning, and inflammatory infiltration (Figure S5C). Sirius red staining showed that KP1 attenuated collagens deposition in the liver (Figure 5G and H). Immunohistochemical staining and Western blotting showed a reduced protein expression of α-SMA, fibronectin, collagen I, and vimentin (Figure 5I-L and Figure S5D). Hepatic mRNA levels of *Acta2*, *Col1a1*, *Mmp7*, *Il1b*, *Il6*, *Tnfa*, *Mcpt1*, *Nos2* genes were suppressed by KP1 (Figure 5M-T). In brief, these results indicate that KP1 ameliorates hepatic fibrotic lesions and inflammation in CCl_4_-induced hepatic fibrosis model.

### KP1 ameliorates cholestatic fibrosis induced by bile duct ligation

**Figure 6:**
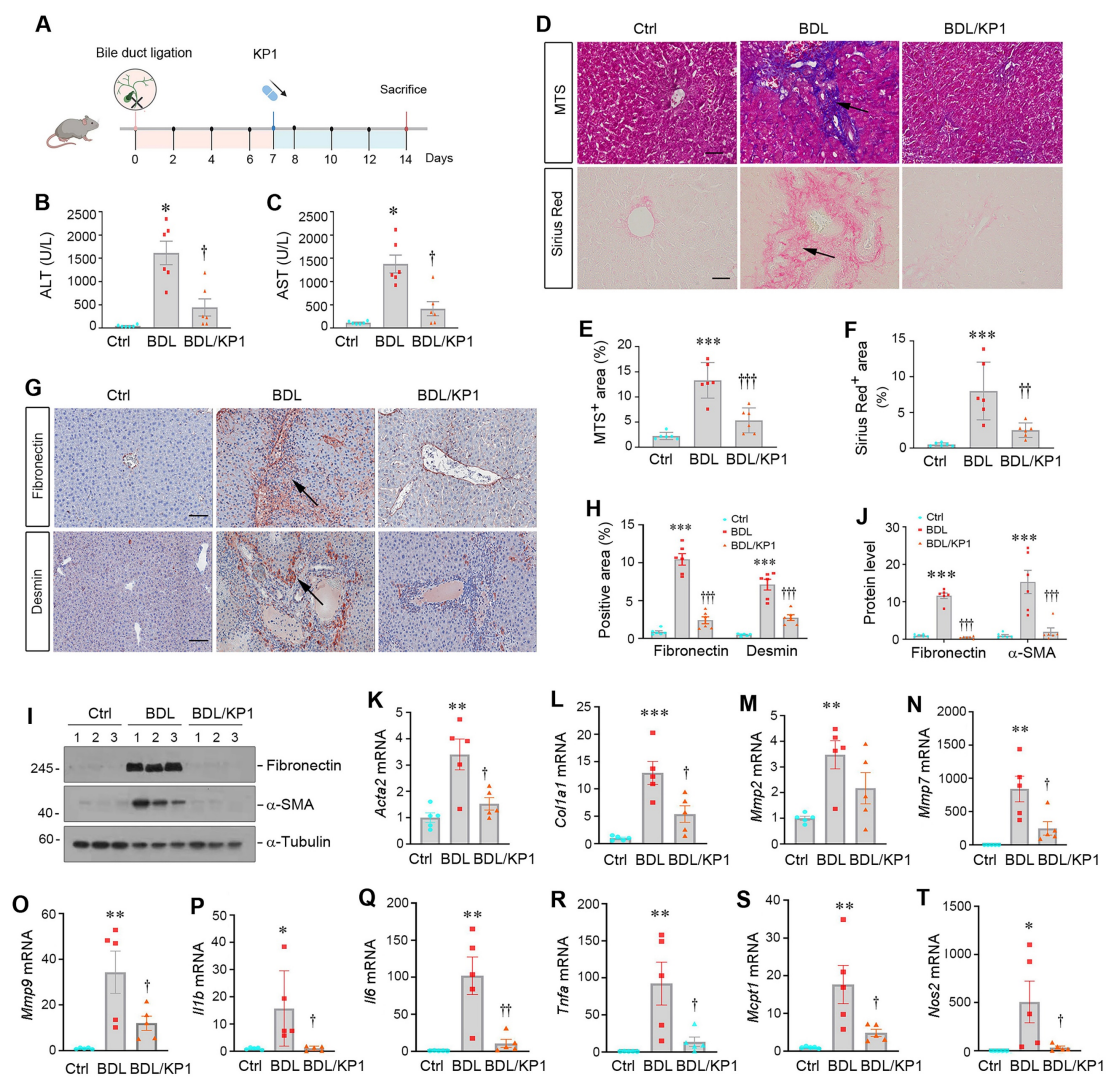
KP1 ameliorates bile duct ligation-induced liver injury and fibrosis in mice. (A) Experimental design. Mice were subjected to bile duct ligation (BDL) to induce experimental liver fibrosis. After 7 days, mice were randomly divided into KP1 (1 mg/day/kg body weight) or vehicle (0.01M acetic acid) groups. The pink circle indicates the BDL surgery. The blue color indicates the duration of KP1 infusions. Mice are sacrificed on day 14. (B-C) Serum ALT and AST levels in BDL mice with or without KP1 treatment. (D-F) Representative micrographs (D) and quantitative data (E, F) of Masson's trichrome staining and Sirius Red staining. Arrows show fibrotic area. Scale bar, 50 µm. (G) Immunohistochemical staining for fibronectin and desmin in the liver section of different groups as indicated. Arrows indicate positive staining areas. Scale bar, 50 µm. (H) Quantitative data of fibronectin and desmin positive areas. (I, J) Representative Western blot (I) and quantitative data (J) show hepatic expression of fibronectin and α-SMA in different groups as indicated. (K-O) KP1 inhibited hepatic expression of multiple fibrosis-related genes. The mRNA expression of *Acta2, Col1a1, Mmp2, Mmp7* and* Mmp9* was assessed RT-qPCR. (P-T) KP1 inhibited hepatic mRNA expression of proinflammatory cytokines. The mRNA expression of *Il1b, Il6, Tnfa, Mcpt1* and *Nos2* was assessed by RT-qPCR. ^*^P < 0.05, ^**^P < 0.01, ^***^P < 0.001 versus controls; ^†^*P* < 0.05 versus, ^††^*P* < 0.01, ^†††^*P* < 0.001 versus BDL (n=5-6).

To generalize the findings that KP1 ameliorates liver fibrosis, we employed another model induced by BDL, which develops cholestatic fibrosis. As shown in Figure 6A, KP1 was administered at 7 days after BDL through intravenous injections. We found that KP1 reduced serum ALT and AST levels after BDL surgery (Figure 6B and C). HE staining indicated that hepatic damage and inflammatory infiltration in BDL liver, which was mitigated by KP1 treatment (Figure S6A). Masson's trichrome and Sirius red staining showed reduced collagens accumulation in KP1-treated BDL mice, compared with BDL-only group (Figure 6D-F). Immunostaining and Western blotting revealed a decreased expression of fibronectin, desmin, vimentin, and α-SMA expression in BDL livers after KP1 treatment, compared with BDL-only group (Figure 6G-J and Figure S6B). RT-qPCR analysis also confirmed that KP1 suppressed the mRNA expression of *Acta2*, *Col1a1*, *Mmp2*, *Mmp7*, *Mmp9*, *Il1b*, *Il6*, *Tnfa*, *Mcpt1*, and *Nos2* genes in the livers after BDL (Figure 6K-T). These results suggest that KP1 inhibits the expression of profibrotic and proinflammatory genes in BDL model as well.

### KP1 blocks TGF-β signaling by disrupting TGF-β1/TβR2 engagement

**Figure 7:**
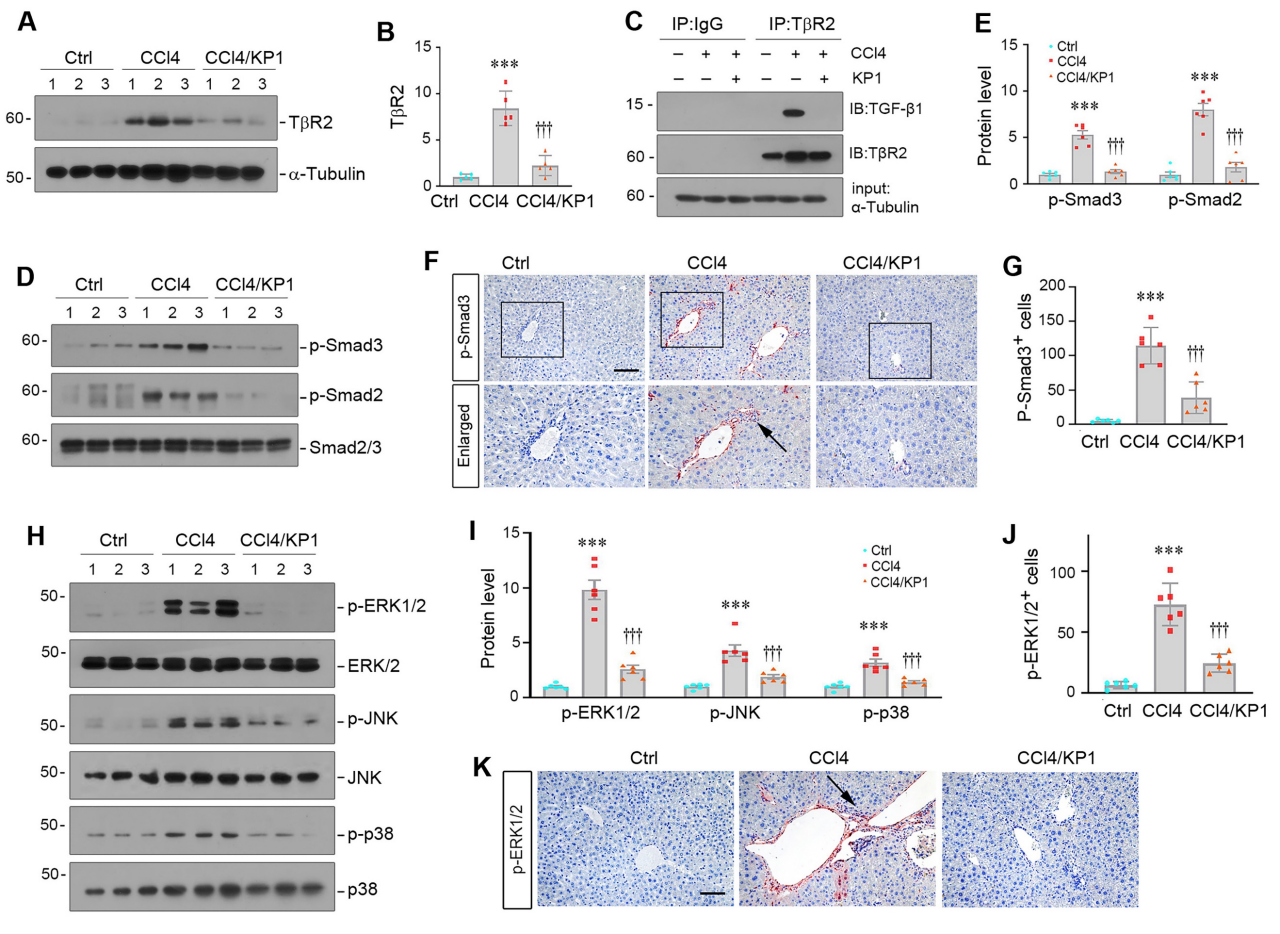
KP1 blocks TGF-β signaling activation in CCl_4_-treated liver. (A, B) KP1 suppressed TβR2 expression in the fibrotic liver induced by CCl_4_. Representative Western blot (A) and quantitative data (B) are shown. (C) Co-immunoprecipitation assay showed that KP1 abolished the binding of TGF-β1 to its TβR2 *in vivo*. Liver lysates were immunoprecipitated with anti-TβR2 antibody, followed by immunoblotting with antibodies against TGF-β1 and TβR2, respectively. (D-E) KP1 inhibited Smad2 and Smad3 activation *in vivo*. Representative Western blot (D) and quantitative data (E) showed the protein levels of p-Smad2 and p-Smad3 in different groups as indicated. (F, G) Representative micrographs (F) and quantitative data (G) showed immunohistochemical staining for p-Smad3. Boxed areas are enlarged. Arrows indicate positive staining. Scale bar, 50 µm. (G) Quantitative data of p-Smad3 positive cell number. (H-I) KP1 inhibited MAPK activation *in vivo*. Representative Western blot (H) and quantitative data (I) showed the protein levels of p-ERK1/2, p-JNK, and p-p38 MAPK, comparing to total ERK1/2, JNK, and p38, respectively. (K, J) Representative micrographs (J) and quantitative data (K) showed the immunohistochemical staining for p-ERK1/2. Arrows indicate positive staining. Scale bar, 50 µm. ^***^P < 0.001 versus controls; ^†††^*P* < 0.001 versus CCL_4_ alone (n=5-6).

We further investigated the mechanism by which KP1 inhibits liver fibrosis *in vivo*. As shown in Figure 7A and B, KP1 reduced hepatic TβR2 expression in CCl_4_ model. More importantly, we found that KP1 disrupted TGF-β1/TβR2 engagement *in vivo*, as evidenced by Co-IP studies (Figure 7C). As a result, KP1 treatment blocked Smad2/3 phosphorylation and activation (Figure 7D-E). Immunohistochemical staining confirmed a reduced nuclear p-Smad3 staining in the liver of CCl_4_-treated mice (Figure 7F-G). Furthermore, KP1 also inhibited the phosphorylation and activation of ERK1/2, JNK, and p38 MAPK in fibrotic livers induced by CCl_4_ (Figure 7H-I). Immunohistochemical staining for p-ERK1/2 also validated a repressed ERK1/2 activation by KP1 in the liver of CCl_4_-treated mice (Figure 7J-K).

**Figure 8:**
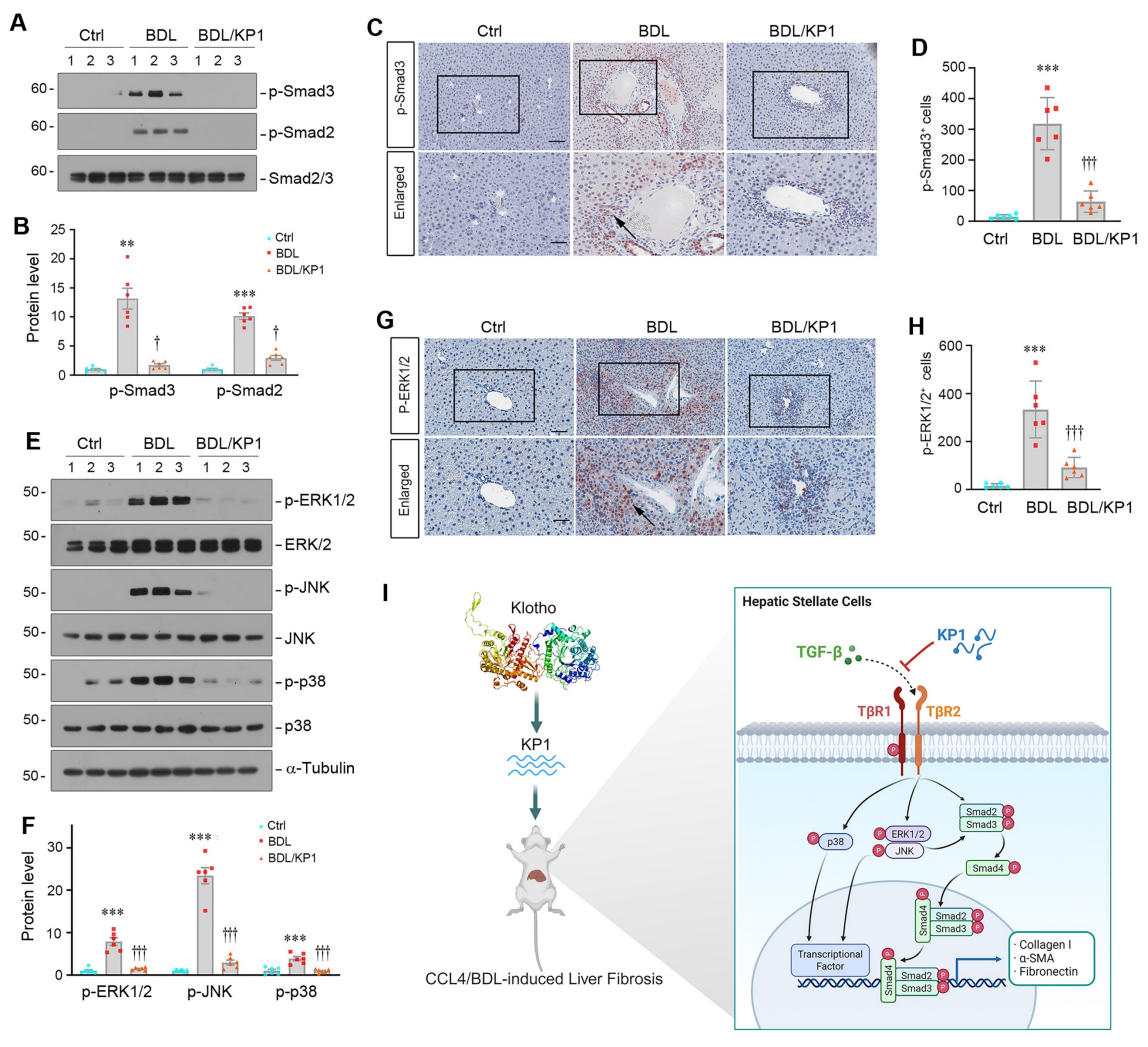
KP1 inhibits TGF-β signaling activation in BDL-induced fibrotic liver. (A, B) Representative Western blot (A) and quantitative data (B) showed p-Smad2 and p-Smad3 protein expression in different groups as indicated. (C, D) Representative micrographs (C) and quantitative data (D) showed the immunohistochemical staining of p-Smad3 in the liver of different groups as indicated. Boxed areas are enlarged. The arrow indicates the nuclear staining of p-Smad3. Scale bar, 50 µm. (E-F) Representative Western blot (E) and quantitative data (F) showed the protein levels of p-ERK1/2, p-JNK, and p-p38 MAPK, comparing to total ERK1/2, JNK, and p38, respectively. (G, H) Representative micrographs (G) and quantitative data (H) showed the immunohistochemical staining for p-ERK1/2. Boxed areas are enlarged. Arrows indicate positive staining. Scale bar, 50 µm. ^**^P < 0.01, ^***^P < 0.001 versus controls; ^†^*P* < 0.05, ^†††^*P* < 0.001 versus BDL alone (n=6). (I) Schematic diagram shows that KP1 attenuates liver fibrosis by inhibiting TGF-β signaling.

Similar inhibition of Smad2/3 phosphorylation and activation was observed in the liver of BDL mice after KP1 treatment (Figure 8A-D). KP1 also inhibited ERK1/2, JNK, and p38 MAPK activation in this model (Figure 8E-H). Taken together, KP1 ameliorates liver fibrosis by disrupting TGF-β/TβR2 engagement, leading to blockage of TGF-β-mediated Smad2/3 and MAPK activation (Figure 8I).

## Discussion

Liver fibrosis, driven by hyperactive TGF-β-mediated HSCs activation, is the common outcome of chronic liver disease but lacks targeted therapies 29-31. In this study, we demonstrate a critical role of αKlotho in constraining TGF-β-driven hepatic fibrosis and present KP1 as a novel inhibitor of liver fibrosis by specifically targeting TβR2 signaling. We show that aging or genetic αKlotho deficiency (*kl/kl* mice) triggers spontaneous hepatic fibrosis via unrestrained canonical TGF-β/Smad and noncanonical TGF-β/MAPK signaling (Figure 1 and S1), positioning the extrahepatic αKlotho as a physiological brake on liver fibrosis. Furthermore, we demonstrate that KP1, a Klotho-derived peptide that recapitulates the anti-fibrotic action of αKlotho, functions as a competitive TβR2 antagonist that suppresses both canonical Smad2/3 and non-canonical MAPK pathways of TGF-β in HSCs, leading to alleviation of the fibrotic lesions in the liver across different preclinical models, including genetic αKlotho-deficient *kl/kl* mice, toxic (CCl₄), and cholestatic (BDL) injury models (Figure 8I). Of interest, KP1 after intravenous injection exhibits highly selective accumulation in the injured liver (Figure 5), underscoring a virtually targeted delivery. These findings offer mechanistic insights into how aging increases susceptibility to liver fibrosis. Importantly, our studies open new avenue for developing effective therapeutics for fibrotic liver diseases, which impact millions of patients worldwide.

HSCs activation is orchestrated by a milieu of mediators, including growth factors, cytokines, and other extracellular cues 4, 9, 32. Among them, TGF-β undoubtedly is the most potent and important one. As a pleiotropic hormone, soluble αKlotho is known to protect against kidney fibrosis through its antifibrotic, anti-inflammatory and antioxidant actions 33, 34. Our discovery that αKlotho deficiency mice (*kl/kl*) develop spontaneous hepatic fibrosis, driven by TGF-β hyperactivation and unchecked collagen deposition, extends this paradigm. This mirrors clinical observations linking low serum αKlotho to more severe tissue fibrosis in multiple organs in patients 35-37. Furthermore, naturally aging mice at the age of 21 months are in the αKlotho-deficient state and also develop spontaneous liver fibrosis (Figure S1), further strengthening this notion. Mechanistically, we demonstrated that αKlotho deficiency disrupts the natural checkpoint on TGF-β signaling, permitting uncontrolled Smad2/3 and MAPK activation (Figure 1). It should be pointed out that αKlotho is not expressed in the liver, but mainly expressed in the kidney (Figure S2). Liver only express βKlotho, a co-receptor for FGF19 and FGF21 that primarily regulates metabolic pathways like bile acid synthesis and energy expenditure 38, 39, which is not altered in αKlotho-deficient *kl/kl* mice. Therefore, αKlotho deficiency due to either genetic ablation or aging causes spontaneous liver fibrosis in a βKlotho-independent manner. These findings underscore that extrahepatically derived αKlotho play a crucial role in restraining TGF-β signaling and hindering tissue fibrosis in the liver.

This study demonstrates an enhanced activation of TGF-β signaling in the liver of *kl/kl* mice at 8 weeks of age, whereas Wnt/β-catenin and FGF2 signaling pathways are unaffected (Figure S3). As these mice get older, it is possible that the Wnt/β-catenin and FGF2 signaling may become activated. Notably, αKlotho is known to modulate insulin and insulin-like growth factor-1 (IGF-1) signaling, both of which are implicated in hepatic metabolism and fibrogenesis 40. Insulin resistance is a hallmark of aging and metabolic syndrome, and is linked to the progression of metabolic dysfunction-associated steatotic liver disease (MASLD) and fibrosis 41. Although not directly examined in the present study, it is plausible that dysregulation of insulin/IGF-1 signaling may contribute to the hepatic phenotype observed in *kl/kl* mice as well. Future studies are warranted to dissect the relative contributions of these metabolic pathways in αKlotho-deficient models and to determine whether KP1 exerts any modulatory effects beyond TGF-β antagonism.

One of the major findings in this study is the identification of key amino acids mediating the interaction between KP1 and TβR2 (Figure 2). Given the pivotal role of TGF-β in HSCs activation and excessive ECM deposition, the ability of KP1 to directly antagonize TβR2 represents a precise and targeted approach for inhibiting TGF-β signaling 15, 42, 43. While αKlotho has been known to interact with TβR2, the structural determinants underlying this binding were previously undefined. Through systematic screening of αKlotho-derived peptides, we identified KP1 (Phe57-Gly86) as the domain conferring high-affinity TβR2 binding 19. The present study through site-directed mutation screening has pinpointed Tyr74 and Gln75 in human αKlotho sequence as critical residues for both binding to TβR2 and functional inhibition (Figure 2), thereby providing a rational framework for future optimization. Functionally, KP1 not only rescued hepatic integrity, restored liver function and attenuated fibrosis in αKlotho-deficient *kl/kl* mice, demonstrating its ability to mimic endogenous αKlotho, but also mechanistically validating TβR2 blockade as its primary antifibrotic action. Several lines of evidence strongly support HSCs as the primary cellular target of KP1. Firstly, KP1 directly inhibits TGF-β1-induced activation, Smad phosphorylation, and pro-fibrotic genes expression in LX-2 cells (Figures 2 and 3). Secondly, the *in vivo* Co-IP assay confirmed that KP1 disrupts the TGF-β1/TβR2 interaction within the fibrotic liver (Figure 7C), a pathway central to HSCs activation. Third, co-localization of Cy5-KP1 with specific myofibroblast marker (α-SMA) in liver sections confirmed the cell-type specificity of KP1 action (Figure 5E). Collectively, these results establish KP1 as a potent, structurally-informed inhibitor of TGF-β-driven fibrogenesis and highlight its promise as a next-generation therapeutic agent.

Another interesting finding in this study is that KP1 is preferentially accumulated in the fibrotic liver, which represents a significant translational breakthrough. The exact reason behind KP1 targeting to injured liver is unknown (Figure 5), but this tropism is likely driven by the upregulation of TβR2 in the injured liver, effectively enabling KP1 to act as a self-guided delivery agent. This tissue-targeting specificity marks a substantial advancement over traditional systemic TGF-β inhibitors, which are often hampered by dose-limiting toxicities due to poor selectivity. Furthermore, the robust efficacy of KP1 across multiple etiologically diverse models, including genetic (*kl/kl*), toxic (CCl₄), and cholestatic (BDL) liver fibrosis, demonstrates its broad therapeutic potential and versatility. Notably, the presence of KP1 in the liver and kidneys of control mice in the absence of CCl₄ treatment is not surprising, as these organs serve as primary sites for drug metabolism. Future studies should focus on elucidating KP1's long-term pharmacokinetics, comprehensive safety profile, and potential synergy with other therapeutics. Taken together, KP1's targeted specificity and therapeutic efficacy underscore its promise as an innovative therapeutic candidate for fibrotic liver disease.

In summary, we show in this study that αKlotho deficiency due to genetic ablation or aging predisposes to TGF-β-mediated hepatic fibrosis through TβR2-driven Smad/MAPK hyperactivation and excessive ECM production. We demonstrate that KP1, a novel αKlotho-derived peptide, acts as a specific inhibitor with liver-selective delivery that targets TβR2 to block TGF-β signaling, leading to mitigation of liver fibrosis. These findings establish KP1 as a promising therapeutic peptide with significant potential for the treatment of chronic liver disease in humans.

## Materials and Methods

### Peptide synthesis

The Klotho-derived peptide 1 (KP1) and its variants were synthesized by GenScript (Piscataway, NJ) with a purity of > 99%. Peptides were dissolved in 0.01 M acetic acid at 3 µM concentration. The sequence of KP1 was reported previously 19.

### Animal models

Male C57BL/6 mice (8-week-old) and male C57BL/6J aged mice (21-month-old) were obtained from Beijing Vital River Laboratory Animal Technology Company. Klotho-deficient mice (*kl/kl*) with C57BL/6J genetic background were described previously 40. The *kl/kl* mice showed the aging-like phenotypes, including a shortened life span, infertility, arteriosclerosis, mitral annular calcification, skin atrophy, osteoporosis, and pulmonary emphysema. After the newborn *kl/kl* mice reached 3 weeks of age, they were genotyped by PCR analysis of tail DNA. The sequences of PCR primers were presented in supplementary Table S1. All mice were housed under specific pathogen-free conditions in accredited animal facilities at Southern Medical University. Animal studies were approved by the Animal Ethics Committee at the Nanfang Hospital, Southern Medical University (NFYY-2022-0523).

At 4 weeks of age, KP1 (1 mg/kg/d) or vehicle were administrated to *kl/kl* mice using ALZET osmotic pumps (Model 1004, DURECT Corporation) for 4 weeks. At 8 weeks of age, mice were killed. The serum, livers, and kidneys were collected for various analyses.

Male C57BL/6 mice (8-week-old) were randomly divided into 3 groups: i) controls; ii) CCl_4_ mice treated with vehicle; iii) CCl_4_ mice treated with KP1. At week 0, liver fibrosis was induced by intraperitoneal injection of CCl_4_ for 8 weeks (0.5 ml/kg body weight, diluted in olive oil, three times per week). Olive oil injection was used as a vehicle control. KP1 peptide was dissolved in 0.01 M acetic acid. KP1 or vehicle was administered continuously via osmotic pumps at the concentration of 1 mg/kg/day starting from week 4. After 48 h of the final CCl_4_ injection, the mice were sacrificed and blood, liver tissues, and kidneys collected for subsequent experiments.

Male C57BL/6 mice (8-week-old) were randomly divided into 3 groups: i) sham controls; ii) BDL mice treated with vehicle; iii) BDL mice treated with KP1. The BDL model was established according to routine protocol as described previously 44. Briefly, under general anesthesia, mice were made a midline abdominal incision. The common bile duct was ligated twice with 6.0 silk sutures and cut through between the ligations. Sham-operated mice were subjected to laparotomy without BDL. The mice were treated with KP1 by intravenous injection at the concentration of 1 mg/kg/day starting from week 1. At week 2, mice were sacrificed and blood and liver tissues collected for analyses.

### Implanting ALZET osmotic pumps

ALZET osmotic pumps had a volume of 100 µl and a release rate of 0.11 µl per hour. Based on the intended administration dosage of KP1, the drug concentration was calculated as 7.576 mg/ml. Using a 1 ml syringe with a blunt needle, KP1 was injected perpendicularly until slight efflux. Subsequently, the pumps were then immersed in sterile PBS and equilibrated at 37°C for 36 h. Under general anesthesia, mice were positioned in the prone position for surgery. The fur was depilated over an area located 0.5 cm caudal to the neck, and the skin was disinfected with alcohol. A horizontal incision about 1 cm in size was made. Then the forceps were used to grasp and insert the prepared pump into the subcutaneous pocket. Finally, the incision was sutured, and the mouse was recovered on a 37 °C heated pad. Upon regaining consciousness and resuming normal activity, the mouse was returned to its clean cage. The pump released continuously for 4 weeks.

### Cell culture and treatment

Human hepatic stellate cell line (LX-2) was obtained from the American Type Culture Collection (Manassas, VA). LX-2 cells were cultured at 37 °C as previously reported 45. After 24 h of serum starvation, LX-2 cells were pretreated with different peptides (3 µM) for 1 h and then incubated with TGF-β1 (#240-B; R&D Systems) at 2 ng/ml. Cells were collected at 45 min or 24 h after TGF-β1 treatment, respectively. After 24 h of serum starvation, LX-2 cells were pretreated with KP1 (3 µM) or sKlotho (100 ng/ml) for 24 h. Whole-cell lysates were prepared and subjected to Western blot analyses.

### Western blot analysis

Western blot analysis was performed as previously described 28. The primary antibodies used were as follows: anti-fibronectin, anti-α-SMA, anti-collagen I, anti-p-Smad3, anti-p-Smad2, anti-p-ERK, anti-p-p38, anti-p-JNK, anti-Smad2/3, anti-JNK, anti-ERK, anti-p38, anti-mKlotho, anti-βKlotho (KLB), anti-TβR2, anti-TGF-β1, anti-TGF-β1, anti-α-tubulin, anti-FITC, anti-GAPDH. The sources of antibodies used are listed in Supplementary Table S2.

### Liver function tests

Alanine aminotransferase (ALT), aspartate aminotransferase (AST), total protein (TP) and albumin (ALB) levels were determined by an automatic chemistry analyzer (AU480; Beckman 496 Coulter, Brea, CA). The levels of ALT and AST were expressed as U/L. The levels of TP and ALB were expressed as g/L.

### Molecular docking simulation

The molecular structures of TβR2 were obtained from the RCSB PDB database (PDB ID:1M9Z), and the structures of KP1, KP1-Mutant3, and sKlotho were established based on the homology modeling technique in Discovery Studio 2019. Molecular docking simulation was performed using ZDOCK and RDOCK program in Discovery studio 2019, and the optimal binding conformation was analyzed.

### Co-immunoprecipitation

Co-immunoprecipitation (Co-IP) was performed as previously described 46. Briefly, to determine if KP1 directly interacts with TβR2, LX-2 cell lysates were prepared and incubated overnight at 4 °C with FITC-KP1 (10 µg), anti-TβR2 antibody, and protein A/G plus agarose (sc-2003; Santa Cruz) for 24 h. The immunocomplexes were blotted with antibodies against FITC and TβR2, respectively. Conversely, LX-2 cell lysates were prepared and incubated overnight at 4 °C with FITC-KP1 (10 µg) or FITC (10 µg), anti-FITC antibody, and protein A/G plus agarose for 24 h. Bound proteins were immunoblotted with anti-TβR2 antibody.

To determine whether KP1 can disrupt the interaction between TGF-β1 and TβR2 in dose-dependent manner, cells were treated with TGF-β1 in the absence or presence of different amounts of KP1 as indicated, and TGF-β1/TβR2 interaction was then assessed.

To assess whether Y18/Q19 in KP1 can act as critical amino acids for its binding to TβR2, cells were treated with TGF-β1 either lacking or containing KP1 or KP1-mutant3/4, as specified, the TGF-β1/TβR2 interaction was subsequently assessed.

### Organ imaging of KP1 distribution

Male C57BL/6 mice were subjected to CCL_4_ injections. Four weeks after injection, control or CCl_4_-injected mice were intravenously injected with 400 µl of Cy5-labeled KP1 (5 mg/kg) or vehicle. All mice were sacrificed after 30 min. Major organs including kidneys, heart, liver, lungs and spleen were removed and placed in glass dishes. Organs were exposed to a Bruker FX PRO imaging system equipped with an excitation at 635 nm and an emission at 675 nm, and images were taken with a camera and digitally analyzed. All procedures were conducted in the dark.

### RT-qPCR

Total RNA was extracted from liver tissue. The mRNA was reverse transcribed to cDNA using the GoScript Reverse Transcription System Kit (Promega, Madison). Quantitative, real-time PCR (qPCR) amplification was performed using a GoTaq Green Master Mix kit (Promega). The sequences of specific primers are given in Supplementary Table S3. *Actb* is used as an internal control for mRNA.

### Histology and immunostaining

Paraffin liver sections were prepared by a routine procedure. Liver sections (3 µm thickness) were subjected to Masson's trichrome staining (MTS) for assessing collagen deposition and fibrotic lesions. Quantification of the fibrotic lesion was carried out by Image J software, and at least three randomly chosen images were analyzed per mouse. Immunohistochemical staining was performed with 3 µm liver sections according to the established protocol 47. The antibodies against fibronectin, α-SMA, p-Smad3, anti-vimentin, and p-ERK1/2 were used. Some samples were subjected to immunofluorescence staining, according to procedures described previously 46. Briefly, LX-2 cells were cultured on coverslips and fixed with cold methanol: acetone (1:1) for 15 min. The slides were immunostained with primary antibodies against fibronectin and p-Smad3 overnight and then stained with Cy3-conjugated secondary antibody (Jackson ImmunoResearch Laboratories). For Cy5-KP1 distribution assay, tissue cryosections were observed for fluorescent imaging by laser confocal microscope (Olympus FV3000, Japan). For Cy5-KP1 distribution assay in fibrotic liver, tissue cryosections were immunostained with primary antibodies against α-SMA overnight and then stained with Cy2-conjugated secondary antibody (Jackson ImmunoResearch Laboratories). The sources of antibodies used are listed in Supplementary Table S2.

### Statistical analysis

All data examined were expressed as means ± SEM. Statistical analyses of the data were performed using SPSS 22.0. Comparison between groups was made by t-test when comparing two groups, or one-way analysis of variance (ANOVA) followed by Student-Newman-Kuels test for more than two groups. *P* < 0.05 was considered statistically significant.

### Data availability

All data are available from the corresponding authors upon request. The information and requests for resources and materials should be directed to Dr. Youhua Liu (liuyh@smu.edu.cn).

## Supplementary Material

Supplementary figures and tables.

## Abbreviations

ALT: alanine aminotransferase
AST: aspartate aminotransferase
ALB: serum albumin
TP: total protein
Acta2: α-smooth muscle actin
α-SMA: α-smooth muscle actin
BDL: bile-duct ligation
CCl_4_: carbon tetrachloride
Col I: collagen I
collagen I: collagen type I alpha 1
collagen III: collagen type III alpha 1
TIMP-2: Metalloproteinase 2
MMP-9: Matrix Metalloproteinase-9
IL-1β: interleukin-1β
IL-6: interleukin-6
TNF-α: Tumor Necrosis Factor-α
MCP-1: monocyte chemoattractant protein-1
NOS2: nitric oxide synthase-2
ERK1/2: extracellular signal-regulated kinase-1/2
FN: fibronectin
GAPDH: glyceraldehyde-3-phosphate dehydrogenase
H.E. staining: hematoxylin-eosin staining
HSCs: hepatic stellate cells
TGF-β: transforming growth factor-β
WT: wild type
