# Supplementary material for: Klotho-derived peptide 1 ameliorates hepatic fibrosis induced by αKlotho deficiency and liver injury
Source: Int J Biol Sci. 2026 Jan 8;22(3):1425–39. doi: 10.7150/ijbs.122107 (PMC12837819; doi:10.7150/ijbs.122107)
Supplement: Supplementary file 1 — Supplementary figures and tables. [file ijbsv22p1425s1.pdf]

Klotho-derived peptide 1 ameliorates hepatic fibrosis induced by αKlotho deficiency and liver injury

Huishi Tan[1, 2†], Wenshu Huang[1, 2†], Hanying Luo[1, 2†], Wenjian Min[3], Xiaoyao Zhang[1, 2], Xiaoli Sun[1, 2], Enqing Lin[1, 2], Xue Hong[1, 2], Peng Yang[3], Lili Zhou[1, 2*], Youhua Liu[1, 2*]

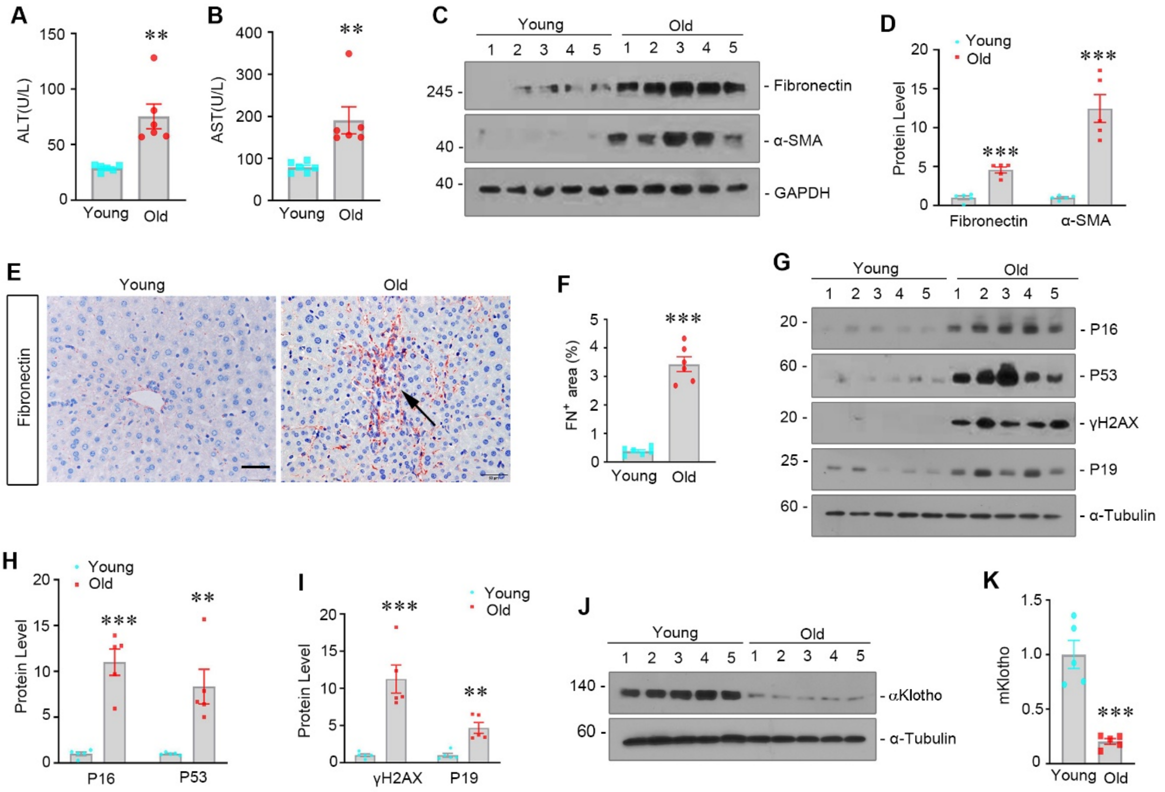

Figure S1. Increased liver injury and hepatic fibrosis is accompanied by reduced αKlotho in aged mice. (A, B) Serum ALT and AST levels in Young (2 months of age) and Old (21 months of age) mice. (C, D) Immunoblot analysis (C) and quantitative data (D) of hepatic expression of fibronectin and α-SMA in Young and Old groups. (E, F) Representative micrograph (E) and quantitative data (F) showed fibronectin expression in the liver section of young and old mice. Arrow indicates positive staining. Scale bar, 50 μm. (G) Immunoblot analysis of hepatic expression of P16, P53, γH2AX, and P19 in young and old mice. (H-I) Quantitative analysis of protein levels of P16, P53, γH2AX, and P19. (J, K) Immunoblot analysis (J) and quantitative data (K) of αKlotho expression in kidney tissues of young and old mice. Data are presented as the mean ± SEM. $^{**}P < 0.01$, $^{***}P < 0.001$ versus young mice (n=5-6).

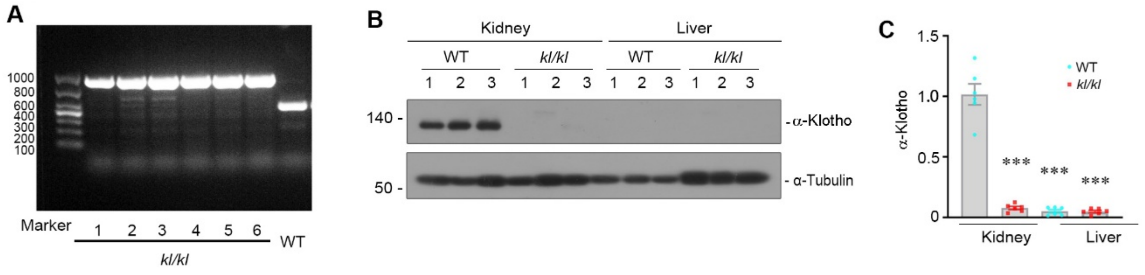

Figure S2. *kl/kl* mice show no expression of α-Klotho in the liver and kidneys. (A) Genotyping was performed using genomic DNA isolated from 3-week-old mice and the sequence-specific primers by PCR. The PCR products were separated using agarose gel electrophoresis to identify PCR products of 458 bp (Wild type) and 920 bp (Homozygotes). (B, C) Immunoblot analysis (B) and quantitative data (C) of renal and hepatic expression of α-Klotho in WT and *kl/kl* mice. Data are presented as the mean ± SEM. $^{***}P < 0.001$ versus WT group in the kidney alone (n=6).

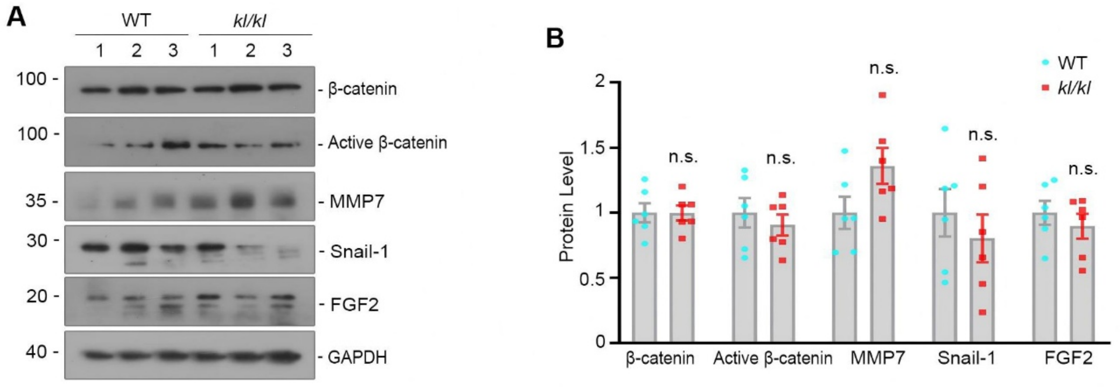

Figure S3. Genetic αKlotho deficiency did not affect hepatic Wnt/β-catenin and FGF2 signaling activation. (A) Representative Western blot images of β-catenin, active-β-catenin, MMP7, Snail1, and FGF2 in the liver of WT and *kl/kl* mice. (B) Quantitative data of β-catenin, active-β-catenin, MMP7, Snail1, and FGF2 in different groups. Data are presented as the mean ± SEM. n.s., not statistical difference (n=6).

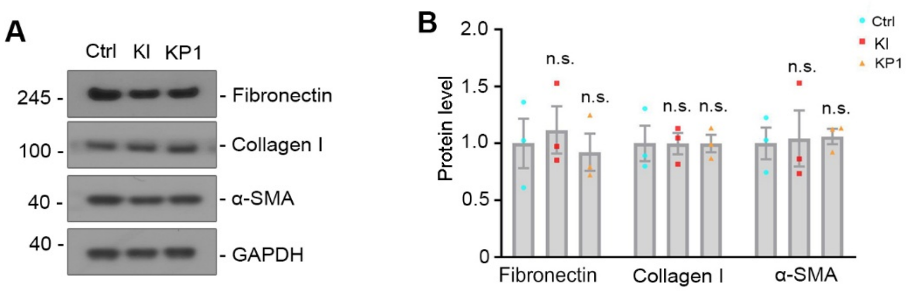

Figure S4. Neither α-Klotho nor KP1 directly regulates HSC activation in the absence of TGF-β1. Representative Western blot (A) and corresponding quantitative data (B) demonstrate that soluble α-Klotho and KP1 did not significantly influence the expression of fibronectin, collagen I, or α-SMA in LX-2 cells in the absence of TGF-β1. Data are presented as the mean ± SEM. n.s., not statistical difference (n=3).

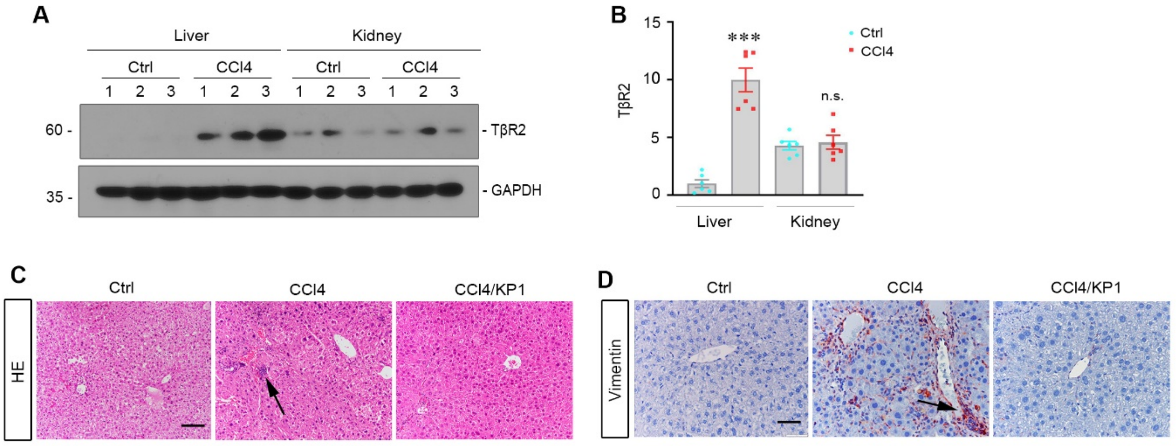

Figure S5. CCl_4-induced liver injury is associated with liver-specific induction of TβR2. (A, B) Immunoblot analysis (A) and quantitative data (B) of renal and hepatic expression of TβR2 in Ctrl and CCl_4 group. Data are presented as the mean ± SEM. n.s., not statistical difference versus Ctrl group in the kidney. $^{***}P < 0.001$ versus Ctrl group in the liver alone (n=6). (C) Representative micrographs of Hematoxylin-eosin staining in the liver of different groups as indicated. (D) Representative micrographs of immunohistochemical staining for vimentin in the liver of indicated groups. Scale bar, 50 µm. Arrow indicates positive staining. Ctrl, control.

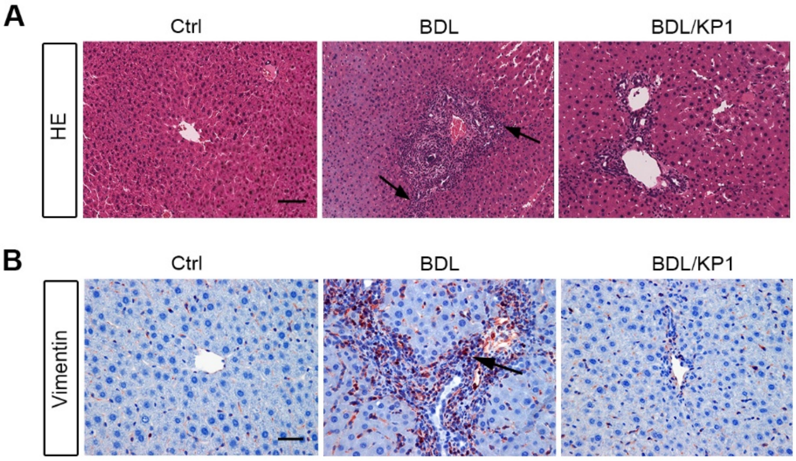

Figure S6. KP1 attenuates bile duct ligation (BDL)-induced liver fibrosis. (A) Representative micrographs of Hematoxylin-eosin staining for BDL-treated liver in different groups as indicated. (B) Representative micrographs of immunohistochemical staining for vimentin in the liver of indicated groups. Scale bar, 50 μm. Arrows indicate inflammatory infiltrates (A) and positive staining for vimentin (B). Ctrl, control.

Table S1. The primer sequence for genotyping *kl/kl* mice

	**Primer sequences**
Primer 1 (common)	TGGAGATTGGAAGTGGACG
Primer 2 (mutant)	CAAGGACCAGTTCATCATCG
Primer 3 (wild)	TTAAGGACTCCTGCATCTGC

Table S2. The detailed information of antibodies used in this study

Name	Manufacturer	Product code	Host	Application
**Primary antibodies**				
Anti-mKlotho	R&D Systems	AF1819	Goat	WB
Anti-KLB (β-Klotho)	Invitrogen	PA5-119246	Rabbit	WB
anti-Fibronectin	Sigma Aldrich	F3648	Rabbit	WB, IHC, IF
anti-α-SMA	Abcam	ab7817	Rabbit	WB
anti-Collagen-1	Boster	BM4017	Rabbit	WB
Anti-p-Smad3	Cell Signaling Technology	#9520	Rabbit	WB, IHC, IF
anti-p-Smad2	Cell Signaling Technology	#3104s	Rabbit	WB
anti-Smad2/3	Cell Signaling Technology	#8685s	Rabbit	WB
anti-TβR2	Santa Cruze Biotechnology	sc-17799	Mouse	WB, IP
anti-TGF-β1	Abcam	ab92486	Rabbit	WB
anti-p-ERK1/2	Cell Signaling Technology	#9101s	Rabbit	WB, IHC
anti-ERK1/2	Cell Signaling Technology	#4695	Rabbit	WB
anti-p-p38	Cell Signaling Technology	#9211s	Rabbit	WB
anti-p38	Cell Signaling Technology	#8690	Rabbit	WB
anti-p-JNK	Cell Signaling Technology	#9251s	Rabbit	WB
anti-JNK	Cell Signaling Technology	#9252	Rabbit	WB
anti-Desmin	Boster	PB9105	Rabbit	IHC
anti-Vimentin	Cell Signaling Technology	#5741s	Rabbit	IHC
anti-FITC	Abcam	ab19224	Goat	WB, IP
anti-α-Tubulin	Beijing Ray Antibody Biotech	RM2007	Mouse	WB
anti-GAPDH	Beijing Ray Antibody Biotech	RM2002	Mouse	WB
**Secondary antibodies**				
Goat anti-mouse	Boster	BA1050	Goat	WB
Goat anti-rabbit	Boster	BA1054	Goat	WB
Rabbit anti-goat	Boster	BA1060	Rabbit	WB
Donkey Anti-Mouse	Jackson ImmunoResearch	715-065-150	Donkey	IHC
Donkey Anti-Rabbit	Jackson ImmunoResearch	711-065-152	Donkey	IHC
Donkey Anti-Goat	Jackson ImmunoResearch	705-065-147	Donkey	IHC
Donkey Anti-Mouse	Jackson ImmunoResearch	715-225-150	Donkey	IF
Donkey Anti-Rabbit	Jackson ImmunoResearch	715-165-152	Donkey	IF

Abbreviations: WB: western blot; IHC: immunohistochemistry; IF: immunofluorescence; IP: immunoprecipitation.

Table S3. The primer sequences of RT-qPCR used in this study

Gene Name	Forward Primer	Reverse Primer
Fn1	GATGAGCTTCCCCAACTGGT	CTGGGTTGTTGGTGGGATOI
Col1a1	GCTCCTCTTAGGGGCCACT	CCACGTCTCACCATTGGGG
Col3a1	CTGTAACATGGAAACTGGGGAAA	CCATAGCTGAACTGAAAACCACC
Mmp2	CAAGTTCCCCGGCGATGTC	TTCTGGTCAAGGTCACCTGTC
Mmp7	CTGCCACTGTCCCAGGAAG	GGGAGAGTTTTCCAGTCATGG
Mmp9	CTGGACAGCCAGACACTAAAG	CTCGCGGCAAGTCTTCAGAG
Timp2	TCAGAGCCAAAGCAGTGAGC	GCCGTGTAGATAAACTCGATGTC
Acta2	GTCCCAGACATCAGGGAGTAA	TCGGATACTTCAGCGTCAGGA
Il6	TGATGCACTTGCAGAAAACA	ACCAGAGGAAATTTTCAATAGGC
Tnfa	CCACCACGCTCTTCTGTCTAC	AGGGTCTGGGCCATAGAACT
Nos2	GTTCTCAGCCCAACAATACAAGA	GTGGACGGGTCGATGTCAC
Il1bβ	ATGCCCACCTCCTCAAAGAC	GTAGTTTCCGTTGGAACAGTGAA
Mcpt1	CCCCAGTCACCTGCTGTTATA	GCTTGGGGTCAGCACAGATC
Actb	CAGCTGAGAGGGAAATCGTG	CGTTGCCAATAGTGATGACC
